# New species of the genus *Cyamops* Melander  from New Zealand  (Diptera, Periscelididae, Stenomicrinae)

**DOI:** 10.3897/zookeys.114.1310

**Published:** 2011-06-30

**Authors:** Wayne N. Mathis, Masahiro Sueyoshi

**Affiliations:** 1 Department of Entomology, PO Box 37012, MRC 169; Smithsonian Institution, Washington, D.C. 20013-7012, USA; 2 Forest Zoology Group, Kyushu Research Center, Forestry and Forest Products Research Institute, 4-11-16 Kurokami, Kumamoto, 860-0862 Japan

**Keywords:** Diptera, Periscelididae (Stenomicrinae), new species, New Zealand

## Abstract

Two new species of the genus *Cyamops* (Diptera: Periscelididae), the first from New Zealand, are described. The two newly described species are: *Cyamops alessandrae* and *Cyamops crosbyi*. A key to the genera of the subfamily Stenomicrinae and to the species of *Cyamops* from the Australasian/Oceanian Region and detailed illustrations of structures of the male terminalia are provided.

## Introduction

The genus *Cyamops* Melander 1913 includes 30 valid species: two from the Afrotropical Region; 12 from the Australasian/Oceanian Region; three from the Nearctic Region, seven from the Neotropical Region, five from the Oriental Region, and one from the Palearctic Region (Mathis and Rung 2011). Previously none was known from New Zealand. The purpose of this paper is to describe two new species from New Zealand that were recently discovered. These discoveries were made while conducting general research on acalypterates of this island nation.

To facilitate identification of these species, we have included a key to the genera of the subfamily Stenomicrinae and to the species of *Cyamops* from the Australasian/Oceanian Region (modified from the key produced by Baptista and Mathis 2000). We have also produced detailed illustrations of structures of the male terminalia of the new species.

## Methods and materials

The descriptive terminology, with the exceptions noted in (Baptista and Mathis (1994, 2000), is that published in the Manual of Nearctic Diptera (McAlpine 1981). The format for the species’ description adheres to (Baptista and Mathis (1994, 2000). Because specimens are small, less than 3.00 mm in length, study and illustration of the male terminalia requires use of a compound microscope. For most of the structures of the male terminalia, we follow the terminology adopted by other workers in Periscelididae (Baptista and Mathis 1994, 2000, Sueyoshi and Mathis 2004). The species’ descriptions are composite and not based solely on holotypes.

Three venational ratios used in the descriptions of new species are based on the largest, smallest, and one other specimen and is defined as: (1) Wing ratio: straight line distance between wing base and apex/greatest straight line distance from anterior margin to posterior margin. (2) 1st costal ratio: the straight line distance between the apices of R1 and R2+3 (costal section II)/distance between the apices of R2+3 and R4+5 (costal section III). (3) 2nd costal ratio: straight line distance between the apices of R2+3 and R4+5 (costal section IV)/distance between the apices of R4+5 and M (costal section III).

Most specimens examined as part of this study are deposited in the New Zealand Arthropod Collection (NZAC, Auckland, New Zealand). A few specimens have been deposited in the Smithsonian Institution (USNM) and California Department of Food and Agriculture (CDFA).

Dissections of male and female genitalia and descriptions were performed using the method of Clausen and Cook (1971) and Grimaldi (1987). Microforceps were used to remove abdomens, which were macerated in a hot sodium hydroxide solution. Cleared terminalia were rinsed in distilled water and 70% ethanol and then transferred to glycerin for observation. For long-term storage, abdomens were placed in an attached plastic microvial filled with glycerin and attached to the pin supporting the remainder of the insect from which it was removed.

## Systematics

### Key to genera of the subfamily Stenomicrinae

**Table:** 

1	Frons with 1 pair of interfrontal setae; eyes bare. Katepisternum with 2 subequal setae. Hindfemur bearing anterodorsal, preapical seta	*Planinasus* Cresson 1914
–	Frons lacking interfrontal setae; eyes microsetulose, sometimes sparsely. Katepisternum bearing 1 prominent seta. Hindfemur lacking anterodorsal, preapical seta	2
2	Fronto-orbital setae reclinate or occasionally mesoclinate, lacking a proclinate seta; medial vertical seta present but with proclinate orientation; face in profile angulate, dorsal surface flattened. Supra-alar seta lacking; lateral scutellar setae 1 pair, apical. Crossvein bm-cu absent, making cells bm and dm confluent; vein CuA2 weak or lacking; cell cu*p* lacking	*Stenomicra* Coquillett 1900
–	Fronto-orbital setae comprising 1 proclinate and 1 reclinate setae; medial vertical seta absent; face in profile shallowly and vertically arched, lacking a flattened, dorsal area. Supra-alar seta present, well developed; lateral scutellar setae variable but usually 2. Crossvein bm-cu well developed, cell bm distinct from dm; vein CuA2 present, well developed; cell cu*p* present	*Cyamops*

### 

#### Genus Cyamops

Melander, 1913

http://species-id.net/wiki/Cyamops

Cyamops Melander 1913: 291. Type species: *Cyamops nebulosus* Melander, by original designation. Sturtevant 1954: 557–559 [revision]. Hennig 1958: 633 [generic characters, relationships], 1969: 610–613 [discussion]. Sabrosky 1958: 169–171 [revision], 1965: 820 [Nearctic catalog]. Khoo 1985: 527–536 [revision, Australian species]. Khoo and Sabrosky 1989: 551 [Australasian/Oceanian catalog]. Baptista and Mathis 1994: 1–25 [revision, New World species], 2000: 481–506 [review]. Grimaldi 2009: 23–27 [revision, Fiji].

##### Description.

Adult: Small flies, body length 1.65–3.30 mm, with slender habitus.

*Head:* Typically dark brown in ground color; fronto-orbits below the insertion of fronto-orbital setae, parafacial and genal region usually with silvery white microtomentum; occipital region shiny, sparsely microtomentose; lower face usually strongly microtomentose below genal region; median plate of clypeus shiny brown; head usually broader than thorax; postcranium strongly concave; eye bearing interfacetal setulae, shape of eye in profile more or less diagonal, lower anterior facets enlarged and encroaching on facial region more in males than in females (males and females of two species from Oceanic Region have the face with almost the same width); eyes closer together at lower edge of frons than at top of head; frons slightly longer than wide; ocellar tubercle small, situated near vertex; mesofrons somewhat depressed, concave; antennal form as for other Periscelididae; arista 3-segmented (see D.K. McAlpine 1983), branched, in some species with basal bifurcate rays; face in profile nearly straight to distinctly angulate, ventral portion (below narrowest gap between eyes) more broadly developed, sometimes shallowly carinate medially, carina narrow to broad, but always broader in females; lower epistomal margin of midface extended around oral opening to form a narrow peristomal area; clypeus a large inverted U, somewhat retracted in males but easily visible in females; palpus short, compressed, with silver luster when viewed under certain angles. Chaetotaxy: Lateral vertical seta slightly to strongly divergent, curved outward; medial vertical and postocellar setae lacking; 2 pairs of fronto-orbital setae, inner pair reclinate, sometimes oriented outward, outer pair proclinate, in most cases pointed inward; inner fronto-orbital seta usually about 3/4 length of outer, rarely shorter; fronto-orbits typically bearing small setulae below setae; true vibrissa apparently absent, but uppermost pair of facial setae developed as porrect, anaclinate “pseudovibrissae,” followed by a row of peristomal setulae and setae at margin of gena; pseudovibrissae sometimes placed externally to row of peristomal setae.

*Thorax:* Shiny, brown to dark brown, sparsely whitish microtomentose, microtomentum more dense at lower portion of katepisternum; calypter brown; postscutellum developed; greater ampulla convex; prosternum bare. Chaetotaxy: Acrostichal setulae in 2 more or less regular rows, slightly diverging behind, being strongly reduced in some species; dorsocentral setae 0+1 or 0+2, 1st seta, when present smaller, 1 row of dorsocentral setulae in front of setae; some setulae also present between acrostichal and dorsocentral rows in some species; supra-alar seta 1, preceded by small setulae, which can be sometimes strongly reduced; scutellum typically with 1 or 2 pairs of setae, basal pair, when present, smaller, notopleural setae 1+1, 1st seta usually longer; katepisternal seta 1, located dorsally (sometimes a 2nd, smaller and weaker seta is present); anepisternum bare or with 1 seta and few setulae along posterior margin. Wing: Costal vein without true costal breaks, but with a weakness before end of vein R1; costal vein extended to vein M; crossvein bm-cu either present, delimiting discal cell from cell bm (species in Afrotropical, Australian, Nearctic, Neotropical, Oriental, and Palearctic Regions), or absent (some Oceanian species); anal vein sometimes strongly reduced in length; anal cell present, well delimited. Legs: Posteroventral setulae of forefemur sometimes differentiated as a row of distal small, spine-like setulae; mid tibia bearing an apicoventral spine.

*Abdomen of Male:* Sparsely microtomentose, brown to dark brown; 6th tergite somewhat narrowed, asymmetrical, extended more on right side near 6th sternite; 7th tergite narrow, asymmetrical, fused to 6th and 7th sternites on left side; 7th right spiracle, sometimes also the left, in 7th tergite; surstyli articulated with epandrium, asymmetrical (left usually longer), connected with hypandrium posteriorly by a weak membrane; cerci small, weakly sclerotized to completely membranous, bearing some setae on posterior half; hypandrium asymmetrical, expanded posteriorly on each side into convex, bowl-shaped structures, sometimes bearing a ventral projection on left side, visible near base of 6th sternite (“hypandrial projection”); parameres apparently absent (a single, setulose postgonite in *Cyamops nebulosus*);gonopods present; aedeagal apodeme long, free or joined posteriorly with hypandrium; aedeagus completely sclerotized; ejaculatory apodeme developed, variously shaped.

*Abdomen of Female:* Syntergosternite 6 a complete ring (tergite and sternite fused), with spiracle within sclerotization; tergite and sternite 7 either fused (Nearctic, Neotropical, Oceanian species), forming a syntergosternal ring enclosing spiracle, or separate (Australian), with spiracle in sternite; segment 8 with tergite and sternite separate, sternite either free (Nearctic and Neotropical species) or partially fused with 7th (Australian species); 2–4 spherical to oblong spermathecae (2 in Australasian/Oceanian species, 3 in Nearctic species, and 4 in Neotropical species).

##### Discussion.

The Australasian/Oceanian fauna demonstrates variation in characters that are constant in American and Afrotropical species, i.e., sexual dimorphism in the shape of the face (absent in *Cyamops micronesicus* and an undescribed species from Ponape) and presence of crossvein bm-cu. All species, however, have a single pair of dorsocentral setae, and the anepisternum lacks setae along the posterior margin.

##### Key to Australasian/Oceanian Species of Cyamops

**Table:** 

1	Basal aristal rays bifurcate	8
–	Basal aristal rays not bifurcate	2
2	Midface flat throughout; face of male wide, not constricted medially	6
–	Midface bearing a vertical carina (male) or a wide elevated portion (female); face of male narrow, constricted medially	3
3	Femora brown; male with left surstylus broadly curved (Fiji)	*Cyamops femobrunneus* Grimaldi, 2009
–	Femora yellow or apically brown, male left surstylus virtually straight	4
4	Ventral facial triangle yellow medially and laterally (New Zealand)	*Cyamops alessandrae* sp. n.
–	Ventral facial triangle white laterally and whitish yellow medially	5
5	Pedicel and basal flagellomere yellow (Fiji)	*Cyamops fiji* Baptista and Mathis, 2000
–	Pedicel and basal flagellomere bicolored, black dorsally, yellow ventrally (New Zealand)	*Cyamops crosbyi* sp. n.
6	Pseudovibrissa placed externally to the row of peristomal setae; basal scutellar seta about 3/4 or more length of apical seta (Micronesia)	“Ponape” species complex
–	Pseudovibrissa aligned with other peristomal setae; basal scutellar seta about 1/2 length of apical seta	7
7	Mesofacial plate without setae; foretibia and tarsus mostly brown to dark-brown; ocellar tubercle shiny; vertex shiny (Yap)	*Cyamops micronesicus* Baptista & Mathis
–	Mesofacial plate setose between upper peristomal setae; foretibia and tarsus mostly yellow; ocellar tubercle dull microtomentose; vertex dull microtomentose (Ponape)	*Cyamops* “species 3”
8	1st costal ratio 2.3 or more	13
–	1st costal ratio 1.0–1.8	9
9	Comb present on ventral margin of midcoxa (Australia)	*Cyamops pectinatus* Khoo, 1985
–	Comb lacking on ventral margin of midcoxa	10
10	Peristomal setae on mesofacial plate (Australia)	*Cyamops claudiensis* Khoo, 1985
–	Peristomal setae on genal suture	11
11	Basal scutellar seta at most 1/3 length of apical seta; tibia and tarsus of foreleg mostly brown to dark-brown (New Guinea)	*Cyamops papuensis* Baptista & Mathis
–	Basal scutellar seta about 3/4 or more length of apical seta; tibia and tarsus of foreleg mostly yellow to yellowish brown	12
12	Wing hyaline (Australia)	*Cyamops truncatus* Khoo, 1985
–	Wing with a conspicuous brown pattern (Australia)	*Cyamops dayi* Khoo, 1985
13	Forefemora with a ctenidium (Fiji)	*Cyamops femoctenidius* Grimaldi, 2009
–	Forefemora lacking a ctenidium	14
14	5th sternite of male abdomen divided medially (American Samoa)	*Cyamops samoensis* Baptista & Mathis, 2000
–	5th sternite of male abdomen entire	15
15	Legs mostly yellowish; midfemur yellow; wing hyaline (Australia)	*Cyamops australicus* Hennig, 1969
–	Legs mostly yellowish brown to black; midfemur brown apically; wing with a conspicuous brown pattern (Australia)	*Cyamops delta* Khoo, 1985

#### Cyamops alessandrae

Mathis and Sueyoshi sp. n.

urn:lsid:zoobank.org:act:8AA31D3E-BFD0-4D99-8827-683E5036FD3C

http://species-id.net/wiki/Cyamops_alessandrae

Figs 1–4 

##### Description.

Adult body length 1.90–2.60 mm; wing length 2.10–3.00 mm; wing width 0.70–1.10 mm.

Head: Ocellar tubercle polished; shiny spot on vertex large and distinct, extended from ocellus 2/3 distance to eye margin; depressed region of frons deep, velvet. Pedicel brownish on dorsal half, otherwise yellow; basal aristal rays minutely bifurcate apically; basal flagellomere yellow, infuscate dorsally. Face constricted medially by the anteroventral margin of the eyes, expanded into a ventral triangular region below level of pseudovibrissae and bearing a vertical, midfacial, yellow carina, facial triangle microtomentose, yellow to whitish yellow, bordered dorsolaterally with yellow stripe, facial setulae in yellow stripe, some specimens with blackish yellow on ventrolateral margin; gena when viewed anteriorly conspicuously silvery white microtomentose, in lateral view more yellowish tan; labellum and palpus pale yellow; face produced, very shallowly angulate. Chaetotaxy: Inner fronto-orbital setae slightly divergent, slightly smaller than lateral vertical seta; arista bearing 6–7 dorsal, 3 ventral rays; pseudovibrissae oriented dorsally; peristomal setae 6–7.

Thorax: Halter brown yellow to yellow; scutellum triangular, posteroapical angle rounded, orientation of scutellum moderately more elevated than scutum, disk a little convex; postpronotum sparsely microtomentose, subshiny to dull. Chaetotaxy: Dorsocentral setae 0+2, length of anterior seta subequal to posterior seta; mesonotal setulae moderately well-developed; scutellar setae 2, basal seta 1/2 length of apical seta. Wing: Hyaline, slightly fuscous; cells bm and dm separated by crossvein bm-cu; 1st costal ratio 2.6–2.9; 2nd costal ratio 2.2–2.7; wing ratio 0.35–0.36; crossvein bm-cu present. Legs mostly yellow; femora mostly yellow, gradually becoming blackish on apical 1/3; tibiae yellowish, blackish basally and especially apically; apical and subapical tarsomere of each leg blackish brown, 3rd tarsomere brown, basal 2 yellowish.

**Figures 1–4.**
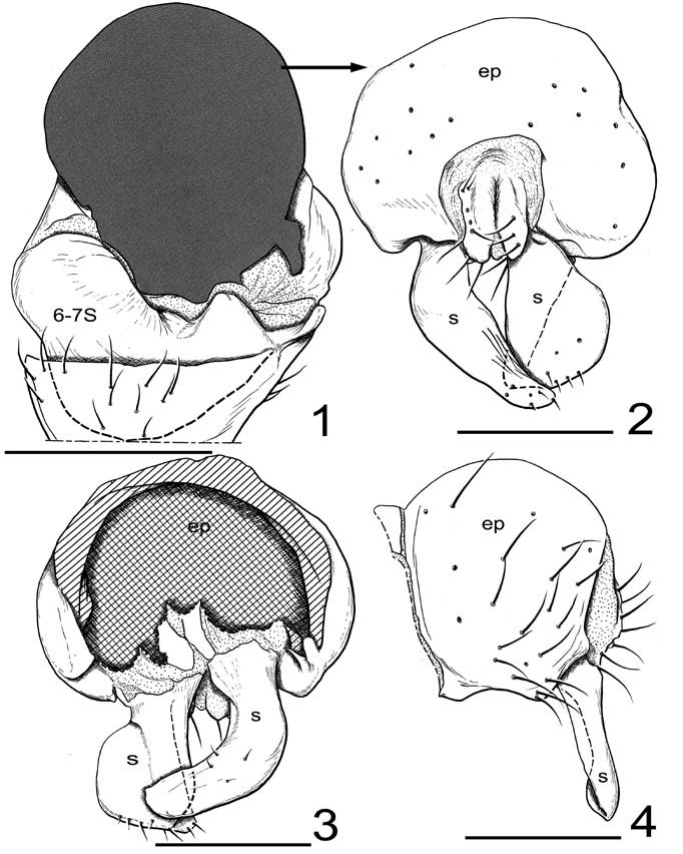
*Cyamops alessandrae* (New Zealand. North Island. WO: Whangamarino Peat Bog). **1** 6th and 7th abdominal segments and postabdomen in ventral view **2** epandrium, proctiger, and surstyli, posterior view **3** same, anterior view **4** same, left lateral view. Epandrium, surstyli, aedeagus, and hypandrium are masked by black tone in Fig. 1. All setae on the epandrium are abbreviated in Fig. 2 and 3. Abbreviations: ep, epandrium; s, surstylus; 6–7S: 6th and 7th abdominal sternites. Scale bar: 1 = 0.20 mm, 2–4 = 0.12 mm.

Male abdomen (Figs 1–4): 6th tergite about same width as dorsal portion of 7th tergite, both sclerites almost without setae; 4th and 5th sternites with well-developed lateral setae and a row of setae along posterior margin; 5th sternite twice as wide as long; 6th and7th sternite asymmetrically bilobed, left lobe much larger than medial lobe. Male terminalia (Figs 2–4): right surstylus in posterior view paddle-like with extended portion angulate ventromedially, lateral margin irregularly rounded, bearing setulae ventrally, basal stem narrowed, broadly stem-like; left surstylus elongate, somewhat digitiform, narrower than right surstylus, basal portion vertical, thereafter oriented ventromedially, apex moderately pointed, in lateral view elongate, almost parallel sided, pointed apically.

Female: Head: Ventral midfacial triangle black with silvery white microtomentum; gena densely microtomentose, silver except on facial carina where microtomentum is thin; basal flagellomere infuscate dorsally.

Thorax: Legs with femora and tibiae mostly brown.

Abdomen: 7th tergite and sternite separate; 7th tergite about 3/4 length of 6th tergite; 2 subequal, spherical spermathecae; sclerotized portion of spermathecal duct about 1/5 length of spermatheca.

##### Type material.

The holotype male is labeled “NEW ZEALAND [North Island:] WO Whangamarino Peat Bog [37°20.9'S, 175°06.8'E], malaise [sic] trap[,] 22 Nov-20 Dec 2006[,] C. H. Watts/Site 2: Manuka/Baumea/Empodisma/N.Z. Arthropod Collection, NZAC Private Bag 92170 AUCKLAND New Zealand [yellow]/HOLOTYPE ♂ *Cyamops alessandrae* Mathis and Sueyoshi NZAC [red].” The holotype is double mounted (glued to a paper point), is in fair condition (head partially collapsed), and is deposited in the NZAC. Twelve paratypes (8♂, 4♀; NZAC, USNM) bear the same locality label data as the holotype. Other paratypes are as follows:

NEW ZEALAND. North Island. WO: Kawhia, Taharoa (38°09'S, 174°44'E; Malaise trap), 22 Nov-20 Dec 2006, C. H. Watts (1♀; NZAC); Kopuatai Peat Bog (37°24.1'S, 175°34.1'E; Site 1: Sporodanthus & Site 2: Sporodanthus-Empodisma; Malaise trap), 22 Nov-20 Dec 2006, C. H. Watts (5♂, 4♀; NZAC).

##### Etymology.

The species epithet, *alessandrae*, is a genitive Latin patronym to honor and recognize the numerous contributions of Dr. Alessandra Rung to the study of Periscelididae and to the genus *Cyamops* specifically.

#### Cyamops crosbyi

Mathis and Sueyoshi sp. n.

urn:lsid:zoobank.org:act:B8FE6378-7DB8-4359-82FD-5704324BB908

http://species-id.net/wiki/Cyamops_crosbyi

Figs 5–12 

##### Description.

Adult body length 2.20–2.55 mm; wing length 2.30–2.75 mm; wing width 0.85–1.00 mm.

**Figures 5–12.**
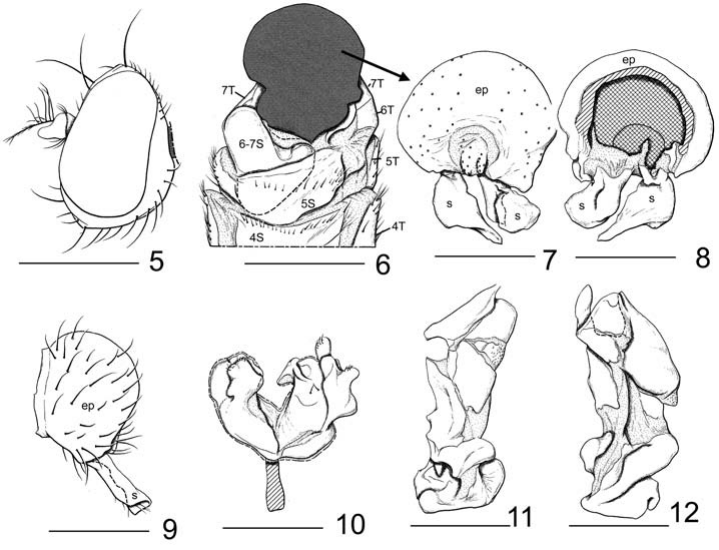
*Cyamops crosbyi* (New Zealand. North Island. AK: Cascade). **5** head, left lateral view **6** male 4–7th abdominal segments and postabdomen, ventral view **7** epandrium, proctiger, and surstyli, posterior view. 8, same, anterior view **9** same, left lateral view **10** hypandrim and gonites in ventral view **11** phallus, left lateral view **12** same, posterior view. Epandrium, surstyli, aedeagus, and hypandrium are masked by black tone in Fig. 6. All setae are abbreviated in Fig. 7. Abbreviations: ep, epandrium; s, surstylus; 4–7S, 4–7T:, 4–7th abdominal sternites and tergites. Scale bar: 5 = 0.50 mm, 6 = 0.40 mm, 7–10 = 0.25 mm, 11–12 = 0.05 mm.

Head (Fig. 5): Ocellar tubercle sparsely microtomentose, subshiny; shiny spot immediately anterior of lateral vertical seta small, about the size of an ocellus; depressed region of frons densely microtomentose, appearing velvety black; fronto-orbits microtomentose, silvery white. Antenna bicolored, dorsal portion brownish black to black, ventral portion yellow; basal aristal rays minutely bifurcate apically; basal flagellomere yellow, infuscate dorsally. Face constricted medially by the anteroventral margin of the eyes, expanding into a triangular region ventrally below the level of the pseudovibrissae and bearing a verticomedial ridge facial region, face yellow in ground color, strongly microtomentose; labellum and palpus pale yellow; face produced and slightly angulate. Chaetotaxy: Inner fronto-orbital setae slightly divergent, slightly smaller than outer vertical seta; arista bearing 9 dorsal, 3 ventral rays, 6 basal rays bifurcate; pseudovibrissae oriented dorsally; peristomal setae 7.

Thorax: Halter brown; scutellum trapezoidal, orientation of scutellum moderately more elevated than scutum, disk a little convex; postpronotum polished. Chaetotaxy: Dorsocentral setae 0+2, anterior seta greatly reduced in length, at most 1/8 length of posterior seta; mesonotal setulae moderately well-developed; scutellar setae 2, basal seta 1/3 length of apical seta. Wing: Hyaline, slightly fuscous; cells bm and dm separated; 1st costal ratio 2.7–3.0; 2nd costal ratio 2.1–2.4; wing ratio 0.35–0.37; crossvein bm-cu present. Legs mostly yellow; femora mostly yellow, gradually becoming blackish on apical 1/3; tibiae yellowish, blackish basally and especially apically; apical and subapical tarsomere of each leg blackish brown, 3rd tarsomere brown, basal 2 yellowish.

Male abdomen (Figs 6–12): 6th tergite about same width as dorsal portion of 7th tergite, both sclerites almost without setae; 4th and 5th sternites with well-developed lateral setae and a row of setae along posterior margin; 5th sternite twice as wide as long; 6th and 7th sternite asymmetrically bilobed, left lobe much larger than medial lobe. Male terminalia (Figs 7–12): right surstylus with extended portion irregularly angulate, in posterior view (Fig. 7) subtriangular, with obtuse angles, basal stem parallel sided basally; left surstylus (Figs 7–9) moderately broad basally, apical half narrow, ventromedial extension, somewhat digitiform, apex pointed, in lateral view elongate (Fig. 9), almost parallel sided, truncate apically; hypandrium and gonites in ventral as in Fig. 10; aedeagus complex, as in Figs 11–12.

Female: Head: Ventral midfacial triangle black with sparse silvery white microtomentum; gena densely microtomentose, silver except on facial carina where microtomentum is thin; basal flagellomere infuscate dorsally.

Thorax: Legs with femora and tibiae mostly brown.

Abdomen: 7th tergite and sternite separate; 7th tergite about 3/4 length of 6th tergite; 2 subequal, spherical spermathecae; sclerotized portion of spermathecal duct about 1/5 length of spermatheca.

##### Type material.

The holotype male is labeled “NEW ZEALAND.N.Isl. AK: Cascade (36°53.2'S, 174°31.2'E; 60 m), 2 Jan 2994[,] Wayne N. Mathis/HOLOTYPE ♂ *Cyamops crosbyi* Mathis & Sueyoshi NZAC [red].” The holotype is double mounted (minuten in a block of plastic), is in excellent condition, and is deposited in the NZAC. Twenty-four paratypes (23♂, 1♀; NZAC, USNM) bear the same locality label data as the holotype.

##### Other material examined.

NEW ZEALAND. AK: Henderson Valley, Scenic Reserve (36°53.8'S, 174°35.7'E; Candia Road entrance. On plants by stream), 14 Jan 2007, S. E. Thorpe (1♀; NZAC).

##### Etymology.

The species epithet, *crosbyi*, is a genitive Latin patronym to honor and recognize the numerous contributions of Dr. Trevor K. Crosby to the study of Diptera from New Zealand, the family Simuliidae in particular.

## Supplementary Material

XML Treatment for Cyamops

XML Treatment for Cyamops alessandrae

XML Treatment for Cyamops crosbyi
